# Supplementary material for: Workflow for high-dimensional flow cytometry analysis of T cells from tumor metastases
Source: Life Sci Alliance. 2022 Jun 3;5(10):e202101316. doi: 10.26508/lsa.202101316 (PMC9166301; doi:10.26508/lsa.202101316)
Supplement: Supplementary file 1 [file LSA-2021-01316_TableS1.docx]

**Supplementary Table 1. Symphony A5 configuration and PMTV setting.** PMTV calibrated by Voltration and Cyto-Cal/QCSB setting procedures are reported as indicated. Cyto-Cal/QCSB and Voltration settings used to generate comparison data are displayed.
Amplifications originally generated have been associated to Rainbow beads fluorescent target values, which have been kept constant over time to neutralize any instrument variation.
