# Supplementary material for: Workflow for high-dimensional flow cytometry analysis of T cells from tumor metastases
Source: Life Sci Alliance. 2022 Jun 3;5(10):e202101316. doi: 10.26508/lsa.202101316 (PMC9166301; doi:10.26508/lsa.202101316)
Supplement: Supplementary file 2 [file LSA-2021-01316_TableS2.docx]

**Supplementary Table 2**. **T cell antibody panel.** The reagents are listed together with their corresponding titrated concentration for 0.5x10^6^ plated cells in 50 μl/sample of Brilliant Stain Buffer (BD). This concentration has to be verified for each batch.
