# Supplementary material for: Workflow for high-dimensional flow cytometry analysis of T cells from tumor metastases
Source: Life Sci Alliance. 2022 Jun 3;5(10):e202101316. doi: 10.26508/lsa.202101316 (PMC9166301; doi:10.26508/lsa.202101316)
Supplement: Supplementary file 3 [file LSA-2021-01316_TableS3.docx]

**Supplementary Table 3**: **T cell antibody panel and titration gate strategy.**

Antibody titrations were evaluated on: ^1^CD3^+^ CD8^+^ HLA-DR^+^ CCR7^-^ cells within total viable ex vivo PBMC; ^2^CD3^+^ CD45RA^+^ cells within total viable ex vivo PBMC; or ^3^total CD3^+^ T cells among viable PHA-activated PBMC (1 μg/ml for 72 h).
